# Endogenous cross-talk of fungal metabolites

**DOI:** 10.3389/fmicb.2014.00732

**Published:** 2015-01-05

**Authors:** Kevin J. Sheridan, Stephen K. Dolan, Sean Doyle

**Affiliations:** Department of Biology, Maynooth UniversityMaynooth, Ireland

**Keywords:** NRPS, gliotoxin, natural products, secondary metabolites, redox homeostasis, siderophores, systems biology, PKS

## Abstract

Non-ribosomal peptide (NRP) synthesis in fungi requires a ready supply of proteogenic and non-proteogenic amino acids which are subsequently incorporated into the nascent NRP via a thiotemplate mechanism catalyzed by NRP synthetases. Substrate amino acids can be modified prior to or during incorporation into the NRP, or following incorporation into an early stage amino acid-containing biosynthetic intermediate. These post-incorporation modifications involve a range of additional enzymatic activities including but not exclusively, monooxygenases, methyltransferases, epimerases, oxidoreductases, and glutathione S-transferases which are essential to effect biosynthesis of the final NRP. Likewise, polyketide biosynthesis is directly by polyketide synthase megaenzymes and cluster-encoded ancillary decorating enzymes. Additionally, a suite of additional primary metabolites, for example: coenzyme A (CoA), acetyl CoA, *S*-adenosylmethionine, glutathione (GSH), NADPH, malonyl CoA, and molecular oxygen, amongst others are required for NRP and polyketide synthesis (PKS). Clearly these processes must involve exquisite orchestration to facilitate the simultaneous biosynthesis of different types of NRPs, polyketides, and related metabolites requiring identical or similar biosynthetic precursors or co-factors. Moreover, the near identical structures of many natural products within a given family (e.g., ergot alkaloids), along with localization to similar regions within fungi (e.g., conidia) suggests that cross-talk may exist, in terms of biosynthesis and functionality. Finally, we speculate if certain biosynthetic steps involved in NRP and PKS play a role in cellular protection or environmental adaptation, and wonder if these enzymatic reactions are of equivalent importance to the actual biosynthesis of the final metabolite.

## INTRODUCTION

Non-ribosomal peptides (NRP) and polyketides are produced by both fungi and bacteria, via non-ribosomal peptide synthesis (NRPS) and polyketide synthesis (PKS), respectively, and are often associated with aiding the organisms to adapt to, or survive in, a hostile environment such as the presence of competing microorganisms, nutrient limitation, and protection against insect immune systems (Meier and Burkart, 2009; Wiemann and Keller, 2014). Although many NRPs and polyketides have known functions [e.g., siderophores (iron acquisition) and antibiotics (bacterial inhibition)] and biomedical applications (e.g., cyclosporin, lovastatin, and mycophenolic acid), the true biological function of many NRPs and polyketides identified to date is unknown, and abrogation of the biosynthesis of certain NRPs, or polyketides, may be without major consequence for the organism (O’Hanlon et al., 2011; Hansen et al., 2012; Steinchen et al., 2013).

With respect to the actual processes, NRPS in fungi is mediated by a combination of large multi-functional enzymes known as NRP synthetases, which include adenylation, thiolation, condensation, and sometimes tailoring domains. NRP synthetases require post-translational modification via 4′-phosphopantetheinylation, mediated by 4′-phosphopantetheinyl transferase (4′-PPTase), to yield the active holo-NRP synthetase. This modification, which requires coenzyme A (CoA) as the 4′-phosphopantetheine source, requires that each thiolation domain within an NRP synthetase is modified at a specific serine residue (Stack et al., 2007). In addition, so-called decorating enzymes or domains are responsible for the modification of NRP biosynthetic intermediates, which may be either tethered to, or released from, the NRP synthetase during the modification process. Likewise, polyketides are biosynthesized from acyl CoA precursors (e.g., malonyl CoA) by multi-modular enzymes consisting of essential ketosynthase, acyl carrier protein and acyltransferase, amongst other, domains (Weissman, 2014). As shown for *Streptomyces* spp. intracellular ATP levels may also affect NRP production in fungi (Li et al., 2008). Unlike PK synthases, NRP synthetases require ATP for NRPS.

Both proteogenic and non-proteogenic amino acids (e.g., ornithine) may be essential precursor substrates for NRP formation and during NRPS are conjugated together generally via peptide bond formation. Moreover, substrate amino acids can be modified either prior to, or during, NRPS (Thykaer and Nielsen, 2003; Chocklett and Sobrado, 2010). In addition, many other cellular components more commonly associated with primary metabolism are required for NRPS and PKS. Amongst others, these include *S*-adenosylmethionine (SAM), isoprenyl, and mevalonyl moieties, nicotinamide adenine dinucleotide phosphate (NADPH), glutathione (GSH), malonyl CoA, and acetyl CoA (Davis et al., 2011; Scharf et al., 2011; Haas, 2012; Yasmin et al., 2012). This suggests significant interplay between what is currently considered to be primary and secondary metabolism and that essential re-consideration must be given to the integration of these two historically defined discrete systems.

In fungi, the biosynthesis of a specific NRP is generally encoded by genes located within a gene cluster, which are often located in the sub-telomeric regions of chromosomes (McDonagh et al., 2008). The activity of these secondary metabolite (SM) gene clusters is controlled by local chromatin structure, which is effected via histone post-translational modification (e.g., methylation or acetylation). The influence of histone modifying enzymes on SM production was first reported for sterigmatocystin (ST) production and gene regulation in *A. nidulans*. The authors uncovered that histone deacetylase mutant Δ*hdaA* bypassed the requirement for the general SM activator LaeA, resulting in overproduction of the subtelomeric metabolites ST and penicillin (Shwab et al., 2007). In the endophytic filamentous fungus *Epichloë festucae* the gene clusters responsible for the production of bioprotective lolitrems and ergot alkaloids were shown to be derepressed following deletion of the genes encoding either the H3K9- (ClrD) or H3K27- (EzhB) histone methyltransferases. A further enhancement of cluster expression was evident when both of these methyltransferases were deleted (Chujo and Scott, 2014). Indeed, the production of many SM in *Aspergillus spp*. is controlled by a methyltransferase, *laeA*, and orthologs of this gene are found in many fungi (Bok and Keller, 2004). Interestingly, LaeA has been shown to counteract the establishment of heterochromatic marks thus activating SM production. Deletion of *laeA* in *A. nidulans* resulted in highly elevated H3K9 methylation levels suggesting that LaeA counteracts H3K9 trimethylation and thus heterochromatin formation at the ST locus (Reyes-Dominguez et al., 2010). The Velvet complex, which is comprised of the light-dependent regulators VeA and VelB, also regulates SM production, and serves to coordinate SM production and fungal development. It is now clear that the VelB–VeA–LaeA complex coordinates fungal sexual development and secondary metabolism (Bayram et al., 2008).

**FIGURE 1:**
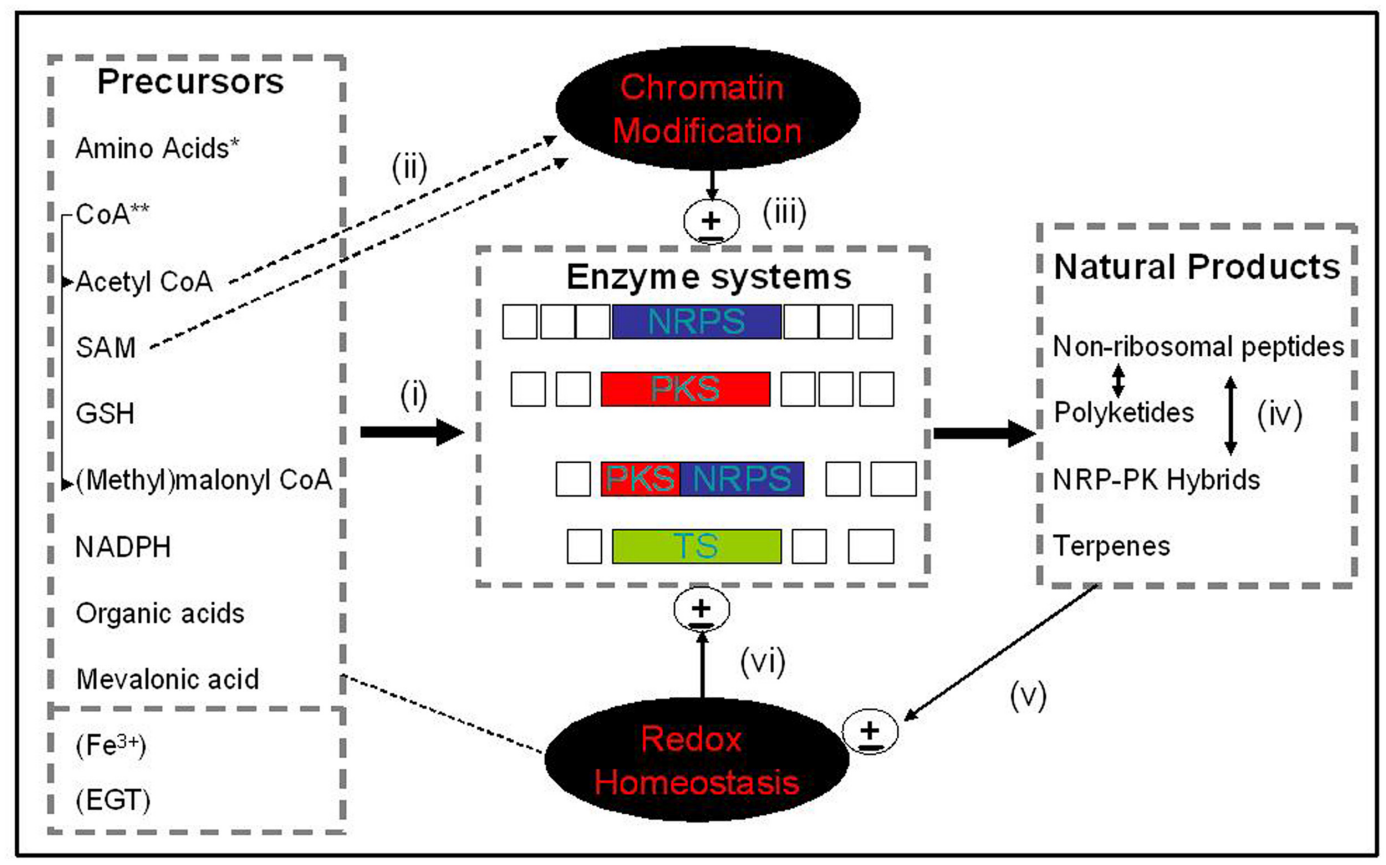
**Systems overview.** An integrated depiction of how precursors from ‘primary metabolism’ (i) feed into enzyme systems [NRPS, PKS, NRPS-PKS, and terpene synthases (TS)] which effect natural product/secondary metabolite biosynthesis and, in part, (ii) affect both chromatin structure (e.g., Acetyl CoA and SAM), and cellular redox status (e.g., GSH, EGT, and Fe^3+^). Chromatin structure, in turn, can (iii) regulate gene cluster expression and metabolite biosynthesis. (iv) Resultant natural products can interact synergistically to enable cellular function (e.g., iron uptake, antioxidant adaptation), or (v) influence cellular redox homeostasis which in turn can (vi) impact on gene cluster expression. Note: Fe^3+^ and EGT are not ’precursors’ *per se*, however, they either bind to (e.g., Fe^3+^-siderophores), or their biosynthesis is influenced by, natural products. *amino acids, proteogenic or non-proteogenic; **CoA, Coenzyme A, a substrate or co-factor for natural product biosynthesis; EGT, Ergothioneine; Open boxes, ancillary enzymes involved in PKS, NRPS, or TS.

Thus, it appears that a truly phenomenal degree of cross-talk exists between so-called primary and secondary metabolism, also involving genetic regulation, to facilitate NRP and polyketide, and indeed other SM, production (**Figure 1**). Primary metabolism is required to provide the essential biosynthetic precursors for NRPS, many NRPs share substrate amino acids and additional co-substrates, chromatin structure apparently controls much SM gene cluster expression, and evidence is also emerging that NRPS pathways interact such that alterations in the biosynthesis of specific NRPs may impact on the production of apparently unrelated metabolites (O’Keeffe et al., 2014; Wiemann et al., 2014). This review is intended to highlight some of the metabolite cross-talk which has been identified to date, and to suggest possible future directions which may further illuminate our understanding of this complex molecular vista- which may, in part, necessitate the development of new models to augment current paradigms of distinct levels of cellular metabolism.

## PRIMARY METABOLITE INVOLVEMENT IN SM BIOSYNTHESIS

### CoA AND ACETYL CoA

It is essential to consider the sources of primary metabolites used during fungal NRPS and PKS in order to begin to see why there may be interactions between apparently independent biosynthetic pathways (**Figure 1**). CoA is somewhat unique, as it functions (i) as a direct substrate which facilitates NRP synthetase and PK synthase activation via 4′-PPTase, and (ii) as malonyl CoA in PKS, and, as acetyl CoA, as the source of acetyl groups for NRPS and histone modification (Stack et al., 2009; Jeon and Lee, 2014; Weissman, 2014). To enable CoA biosynthesis, L-valine and L-aspartate are initially converted to pantoate and β-alanine, respectively, followed by condensation to form pantothenate (vitamin B5), which in turn is converted to CoA by the action of a multi-step biosynthetic pathway involving condensation to ADP (Spry et al., 2008; Chen et al., 2014a; **Figure 1**). Cytosolic acetyl CoA is provided by β-oxidation of fatty acids, from acetate through the action of acetate synthetase (Acs1), and via the ATP-citrate lyase (Acl1) which uses citrate as a substrate (Griffiths et al., 2012). Indeed it has been demonstrated that Acl1 and 2 function to regulate cytosolic acetyl CoA levels in *A. niger* which in turn affects growth and development (Chen et al., 2014a). Thus, it is clear that provision of two essential biomolecules (CoA and acetyl CoA) for fungal NRPS and PKS requires close interplay with primary metabolic processes. Interestingly, although studies directly pertaining to NRPs appear rare, it has been demonstrated that biosynthesis of the polyketide lovastatin in *A. terreus* is enhanced by media supplementation with calcium D-pantothenate, amongst other B vitamins (Bizukojc et al., 2007).

### SAM

*S*-adenosylmethionine is the major source of methyl groups for cellular reactions involving methylation, and is directly synthesized via the action of SAM synthetase (Sauter et al., 2013). In fungi, the methionine cycle enables SAM biosynthesis, whereby L-homocysteine is converted to L-methionine via methionine synthase, SAM is formed in turn via SAM synthetase which requires ATP (Sieńko et al., 2009). Cellular methylation reactions (e.g., NRPS and PKS) then consume SAM to produce *S*-adenosylhomocysteine (SAH), which in turn is converted to L-homocysteine, thereby completing the methionine cycle (Liao et al., 2012). In *A. nidulans*, a SAM synthetase, SasA, has been demonstrated to affect SAM availability which is important for the production of ST, but also may play a role in coordinating fungal secondary metabolism and development (Gerke et al., 2012). These authors demonstrated SasA interaction with histone 2B via TAP-tag technology and LC-MS, and suggested that this interplay may facilitate epigenetic control via methylation. SAM availability is essential for the biosynthesis of a range of polyketides and NRPs including gliotoxin, where it acts as a methyl source for the *N*-methyltransferase GliN (Scharf et al., 2014), and the negative regulator of gliotoxin biosynthesis GtmA, which attenuates gliotoxin biosynthesis via SAM-dependent *bis*-thiomethylation of dithiolgliotoxin (Dolan et al., 2014). SAM is also the provider of methyl groups for the biosynthesis of many other NRPs and polyketides such as butyrolactone III (Guo et al., 2013a). Forseth et al. (2011) identified a plethora of methylated biosynthetic intermediates/shunt metabolites associated with gliotoxin biosynthesis which allowed the authors to speculate about the complex reactions required within this metabolic pathway.

Methionine and SAM are sulfur-containing metabolites, and it has recently been demonstrated that the bZIP transcription factor MetR plays an important role in sulfur assimilation in *A. fumigatus* (Amich et al., 2013). While these authors elegantly demonstrated that MetR functionality was essential for growth on inorganic sulfur sources, it was observed to be less important for regulating genes involved in methionine metabolism. Nonetheless, they identified a key interaction with iron homeostasis, whereby under sulfur-limiting conditions, genes involved in siderophore biosynthesis (*sidA*), siderophore transport (*mirB*), and iron regulation (*hapX*) underwent increased expression in *A. fumigatus* Δ*metR* – although sufficient Fe^3+^ was available in the media. Moreover, significantly elevated levels of the intracellular siderophore, ferricrocin, a NRP, were also observed which was necessary to chelate elevated intracellular Fe^3+^ to prevent cellular damage in Δ*metR*. Given the essential role of Fe–S cluster proteins in energy generation, this regulatory cross-talk, which involved altered iron-sensing due to defective sulfur assimilation, and led to altered NRP [ferricrocin (FC)] levels significantly underpins the integrated nature of cellular metabolism in fungi.

### GSH

The biosynthesis of gliotoxin, acetylaranotin, and related epipolythiodioxopiperazine (ETP) compounds requires biosynthetic intermediate sulfurization and it has been established that GSH is this sulfur source (Davis et al., 2011; Scharf et al., 2011; Guo et al., 2013b). Furthermore, Atanasova et al. (2013) have noted elevated expression of genes predicted to be involved in GSH formation, possibly as a precursor to enable the biosynthesis of gliotoxin in *Trichoderma virens*. In any case, during gliotoxin biosynthesis a gene cluster-encoded glutathione *S*-transferase (GST) mediates GSH conjugation to a highly reactive acyl imine intermediate which results in a *bis*-glutathionylated product, which is subsequently processed by a suite of enzymes, initially by a γ-glutamyl cyclotransferase (GliK in the case of gliotoxin biosynthesis) to the final product (Davis et al., 2011; Gallagher et al., 2012; Scharf et al., 2011, 2013). Thus, a key cellular reductant, which can also undergo oxidation to the GSSG form to maintain cellular redox homeostasis, is an essential substrate for NRP biosynthesis. Indeed, it is interesting to speculate how, and why, fungi utilize an antioxidant (GSH) to generate an alternative metabolite, gliotoxin, with redox-active properties. Perhaps the ability of fungi to effect gliotoxin (and related ETP) but not GSH secretion is in part responsible for this apparent puzzling situation.

### ERGOTHIONEINE

Ergothioneine (EGT) is a sulfurized and tri-*N*-methylated L-histidine derivative, and functions as an antioxidant due to its high redox potential (Fahey, 2013). It is produced by non-yeast fungi, with the gene EGT-1 found in all fungal phyla, except the Saccharomycotina subphylum (Genghof, 1970; Jones et al., 2014). EGT exists as a tautomer between its thione and thiol forms, however, it is predominantly found in the thione form at physiological pH (Carlsson et al., 1974). While most naturally occurring thiols have a redox potential ranging from -0.2 V and -0.32 V, EGT has a redox potential of -0.06 V, which means that it is less susceptible to auto-oxidation compared to GSH (Jocelyn, 1972). In *Neurospora crassa*, ergothioneine has been proposed to protect conidia against oxidative stress (Bello et al., 2012). Although not strictly a NRP, recent work has revealed further requirements for provision of SAM and γ-glutamylcysteine (and molecular O_2_) to enable ergothioneine biosynthesis in Mycobacteria (Seebeck, 2010). In *A. fumigatus*, Gallagher et al. (2012) observed that EGT levels increased significantly when production of the NRP gliotoxin was abrogated. The significance of this correlation, if any, has yet to be elucidated. In other fungi (*Schizosaccharomyces pombe*), the multi-functional enzyme Egt-1, which comprises a methyltransferase domain and a hercynylcysteine sulfoxide synthase activity, requires both SAM and L-cysteine to enable ergothioneine biosynthesis – whereby SAM provides methyl groups and L-cysteine is the sulfur source (Pluskal et al., 2014). As noted, Gallagher et al. (2012) were the first to demonstrate that deletion of *gliK* from *A. fumigatus* not only abrogated gliotoxin biosynthesis, but also resulted in significantly elevated levels of intracellular ergothioneine. Although these authors noted a significantly elevated sensitivity to H_2_O_2_-induced oxidative stress, the precise mechanistic details of the interaction between these redox-active metabolites is presently unclear, and future work is likely to dissect the nature of this cross-talk. However, it is not inconceivable that ergothioneine biosynthesis is increased to, in part, compensate for the abrogation of gliotoxin biosynthesis at a specific biosynthetic step.

### NAPDH

Nicotinamide adenine dinucleotide phosphate requirement as a reductant in NRPS and PKS is well-established (Manavalan et al., 2010; Wilson et al., 2013) and recent work in mammalian cell lines (Fan et al., 2014) has demonstrated that both the oxidative pentose phosphate pathway and one-carbon metabolism are important cellular sources of NADPH. While it has been reported that metabolic engineering of the pentose phosphate pathway – consequent to increased abundance of 6-phosphogluconate dehydrogenase – can lead to enhanced cellular NADPH availability in *A. niger* (R Poulsen et al., 2005), to our knowledge little assessment of alternative sources of NADPH in fungi has been forthcoming. Thus, in future assessments of integrated fungal metabolism, it is possible that relevant interactions between NRPS, PKS, and availability of alternative reducing power require evaluation.

### AMINO ACIDS

A link between amino acid biosynthesis and secondary metabolism has been described in the ascomycetous plant pathogen *Leptosphaeria maculans*. The NRP sirodesmin PL, an ETP, which is the causal agent of blackleg disease of *Brassica napus*, has been shown to be regulated by the cross pathway control gene *cpcA*, which in turn regulates amino acid biosynthesis. During amino acid starvation, amino acids are diverted from sirodesmin biosynthesis into amino acid biosynthesis, and a silenced *cpcA* mutant was shown to produce significantly higher levels of sirodesmin PL after prolonged amino acid starvation compared to the wild-type strain. However, it has yet to be established if this effect is mediated directly or indirectly through the sirodesmin pathway specific transcriptional factor, *sirZ* (Elliott et al., 2011). The rice pathogen *Fusarium fujikuroi* produces the NRP apicidin F under nitrogen-sufficient conditions through the global regulator AreB (Niehaus et al., 2014). Similarly, production of the *F. fujikuroi* PKS/NRPS-derived mycotoxin fusarin C is induced by an excess of nitrogen (Wiemann et al., 2010). Organic acids such as fumarate, anthranilic acid, 4-hydroxyphenylpyruvate, and indole-3-pyruvate also are incorporated into NRPs in fungi (Wackler et al., 2012; Steinchen et al., 2013; Walsh et al., 2013).

### RIBOSOMAL PEPTIDE SYNTHETIC PATHWAY (RiPS)

The discovery of the biosynthetic mechanism, via a ribosomal peptide synthetic pathway (RiPS), for ustiloxin B in *A. flavus* by Umemura et al. (2014) can be thought to further contribute to the blurring distinction between primary and secondary metabolism. Ustiloxin B is a modified YAIG tetrapeptide, conjugated to norvaline via L-tyrosine. However, it appears that YAIG is derived from a proteinaceous precursor, followed by extensive enzymatic conversion, which is encoded by a discrete gene cluster, to ustiloxin B. Of special interest is the presence of a S atom in ustiloxin B, which is coincident with the presence of a GST, cysteine desulfurase and γ-glutamyl transpeptidase in the cognate gene cluster (Umemura et al., 2014). Although not noted by the authors, it is tempting to speculate that a similar biochemistry to that which has evolved to enable ETP biosynthesis in fungi, is also operational in the biosynthesis of a RiPS product, ustiloxin B, in an NRPS-independent manner. This work is the first example of RiPS in Ascomycetes.

## METABOLITES AND NON-PROTEOGENIC AMINO ACIDS – THE ORNITHINE EXEMPLAR

Siderophores are NRPs secreted by fungi and bacteria that scavenge free ferric iron (Fe^3+^) in the immediate environment to facilitate its transport into the cell, and this mechanism is deployed by both animal and plant pathogens (*Cochliobolus heterostrophus*) to acquire iron (Haas, 2012; Zhang et al., 2013). Because Fe^3+^ is required for primary metabolism, it is clear that these NRPs also act as a nexus between both primary and secondary functionalities within fungi. Since excess intracellular Fe^3+^ is deleterious, intracellular siderophores also exist to maintain intracellular Fe^3+^ homeostasis and prevent Fenton reaction oxidative damage (Eisendle et al., 2006). In *Aspergillus spp*. such as *A. nidulans* and *A. fumigatus*, triacetylfusarinine C (TAFC) and fusarinine C (FusC) are secreted to acquire Fe^3+^, while FC is the key intracellular siderophore which effects Fe^3+^ storage. It is notable that in *A. niger*, TAFC is absent and coprogen B and ferrichrome are the main secreted siderophores, with the latter comprising the main intracellular siderophore (Franken et al., 2014). Biosynthesis of siderophores necessitates interaction between enzyme systems at multiple levels. Firstly, one of the key non-proteinaceous amino acids, L-ornithine, essential for siderophore biosynthesis, must be converted to *N*-hydroxy-L-ornithine via the action of ornithine monooxygenase, SidA (Schrettl et al., 2004; Hissen et al., 2005). Interestingly, Beckmann et al. (2013) have demonstrated that mitochrondrially derived ornithine is primarily utilized for siderophore biosynthesis – indicating a localization-type cross-talk. These authors also demonstrated metabolite cross-talk between siderophore and polyamine formation, whereby ornithine acts a co-substrate for SidA and ornithine decarboxylase (ODC), the latter enzyme acting to provide biosynthetic precursors for polyamine biosynthesis. Secondly, mevalonate, a biosynthetic intermediate in ergosterol biosynthesis, is also required for siderophore biosynthesis and is enzymatically converted to anhydromevalonyl-CoA to facilitate incorporation into FusC and TAFC (Schrettl et al., 2007). Finally, in *A. fumigatus*, FusC is converted to TAFC by enzyme-mediated acetylation via the action of SidG in an acetyl CoA-dependent reaction. The cross-talk between ergosterol and siderophore biosynthesis has proven to be especially significant, as it has been demonstrated that inhibition of HMG CoA reductase, the enzyme responsible for mevalonate production (**Figure 1**), also attenuates siderophore biosynthesis, and virulence in a murine ocular keratitis model system, (Leal et al., 2013). Moreover, this inhibitory effect is enhanced by utilization of iron chelators to further inhibit iron uptake by fungi. Thus, interfering with metabolite cross-talk systems may bring therapeutic benefits.

## METABOLITE CROSS-TALK AND OXIDATIVE STRESS

As aerobic organisms, fungi are subject to oxidative stress due to the production of reactive oxygen species (ROS) as side-products of metabolic pathways. Almost 90% of ROS in aerobic organisms is produced through the electron transport chain, while oxidases such as NADPH oxidase also contribute toward the ROS content of the cell (Takemoto et al., 2007; Starkov, 2008). Control of ROS levels is important as cellular components can be modified by ROS, particularly enzymes containing [Fe–S]-clusters and active thiol groups. Oxidative stress, whereby the ROS concentration rises above tolerable levels, thereby leading to deleterious modification of cellular components, can interfere with cellular metabolism and regulatory pathways (Lushchak, 2011). Enzymes such as catalases, glutathione peroxidases, and superoxide dismutases actively protect against oxidative stress, while fungi also produce antioxidant molecules such as α-tocopherols, ascorbic acid, carotene and GSH (Halliwell and Gutteridge, 2007). SM also interacts with ROS in many ways. Some SMs act as antioxidants (EGT, as described above) or otherwise protect against oxidative stress (Bello et al., 2012), while others use oxidative stress as a signal to initiate biosynthesis, while some SMs, like ETPs, enact toxic effects through ROS generation and redox reactions (Gardiner et al., 2005a).

Epipolythiodioxopiperazines are categorized by a signature disulfide bridge (Gardiner et al., 2005a) and gliotoxin is the best studied of the ETPs. It is produced by *A. fumigatus*, and its biosynthesis is mediated by the products of the *gli* gene cluster, with the NRP synthetase GliP starting the pathway through the fusion of L-phenylalanine and L-serine (Balibar and Walsh, 2006), while many of the other genes encode decorating enzymes. Gliotoxin toxicity is intrinsically linked to its disulfide bridge, and redox cycling between the disulfide and dithiol forms allows for the production of ROS. The ability of gliotoxin to form mixed disulfide bonds with proteins, resulting in their inactivation, is another example of the redox-dependant action of the disulfide bridge (Waring et al., 1995). *A. fumigatus* in part detoxifies gliotoxin by closing the disulfide bride via the oxidoreductase GliT, which ‘disarms’ the toxic effects of the molecule, although a cryptic reductase activity of GliT has also been noted (Scharf et al., 2010; Schrettl et al., 2010). Another ETP, sporidesmin, effects toxicity in a similar manner to gliotoxin (Middleton, 1974; Munday, 1982). The redox activity of gliotoxin does not appear to be limited to anti-microbial and virulence-related activity upon secretion and intracellular gliotoxin appears to play a role in maintaining the redox state of the cell. Owens et al. (2014) demonstrated that gliotoxin alleviates oxidative stress caused by H_2_O_2_ exposure in *A. fumigatus* which suggests that gliotoxin is not merely a toxin, but may play an important role in redox homeostasis in *A. fumigatus*. Thus, this SM appears to mediate cross-talk between ROS presence and the global response to ROS in fungi (**Figure 1**).

Reverberi et al. (2010) have also highlighted a link between production of SMs and oxidative stress. Interestingly, oxidative stress in *A. parasiticus* up-regulates aflatoxin biosynthesis (Jayashree and Subramanyam, 2000; Narasaiah et al., 2006; Reverberi et al., 2008). Conversely, aflatoxin biosynthesis is inhibited in response to antioxidant activity (Reverberi et al., 2005). The aflatoxin regulatory gene, *aflR*, encodes a Zn(II)_2_Cys_6_ DNA binding protein serves to activate gene expression encoding enzymes for the ST/aflatoxin biosynthetic pathway (Yu et al., 1996). The discovery of putative binding sites for the Yap1 ortholog, AP1, in the *aflR* promoter region further strengthens this suggested relationship (Reverberi et al., 2008). A similar relationship between oxidative stress and SM production can be seen in *Fusarium graminearum*. Addition of H_2_O_2_ to liquid cultures increased the accumulation of the type B trichothecenes, deoxynivalenol, and 15-acetyl-deoxynivalenol, while the addition of catalases down-regulated expression of the genes responsible for their biosynthesis (Ponts et al., 2006, 2007). Similar results showing antioxidants reducing SM biosynthesis have been encountered in *A. flavus* with aflatoxin (Chipley and Uraih, 1980), *F. verticillioides* with fumonisin B_1_ (Beekrum et al., 2003), *A. ochraceus* with ochratoxin A (Fanelli et al., 2004) and *Penicillium expansum* with patulin (Sanzani et al., 2009; Tolaini et al., 2010). This demonstrates cross-talk between oxidative stress defense and SM biosynthesis across a range of fungi, producing several apparently unrelated SMs.

Siderophores, the iron-scavenging NRPs described above, have an essential role in oxidative defense. Excess intracellular iron can result in oxidative stress through Fenton reaction (Papanikolaou and Pantopoulos, 2005), while conversely, heme is essential for the function of many peroxidases (Conesa et al., 2002), an important family of enzymes in the detoxification of hydrogen peroxide. Thus it is important for fungi to manage iron levels carefully. Eisendle et al. (2006) demonstrated that oxidative stress causes an up-regulation of the intracellular siderophore ferricrocin in *A. nidulans*. Such an increase is also seen when exposed to iron excessive conditions, though it is unclear if this is due to the iron itself, or the resulting iron mediated oxidative stress on the cell. Deletion of the siderophore synthetase *sidC* halts production of ferricrocin, resulting in multiple phenotypes, such as inefficient iron utilization, delayed germination in iron-replete conditions and elimination of cleistothecia formation in homothallic conditions. In addition to these phenotypes, the conidia of the *sidC* mutant display sensitivity to H_2_O_2_. A similar relationship role of siderophores in oxidative defense can be seen in *A. fumigatus*. Schrettl et al. (2007) investigated several siderophore biosynthetic gene deletions. The siderophore null mutant, Δ*sidA* demonstrated severe sensitivity to H_2_O_2_, while absence of either intracellular or extracellular siderophores separately resulted in a milder sensitivity. This suggests that both intra- and extra- cellular siderophores have a role in mediating oxidative damage and suggests redundancy in the system. Cross-talk between ROS detoxification and siderophores is not limited to the *Aspergillus* species. In *Alternaria alternata*, deletion of NPS6, a NRPS essential for extracellular siderophore biosynthesis, results in increases sensitivity to H_2_O_2_. In addition, expression of NPS6 in *A. alternata* is regulated by NOX, YAP1, and HOG1, genes involved in oxidative stress defense (Chen et al., 2014b). Deletion of NPS6 *in Cochliobolus miyabeanus, Fusarium graminearum*, and *Alternaria brassicicola* also resulted in increased sensitivity to oxidative stress (Oide et al., 2006).

Medentsev and Akimenko (1998) conducted a large scale investigation into naphthoquinones, redox active SMs produced by a large variety of filamentous fungi. The study demonstrated that many of these (fusarubin, anhydrofusarubin, javanicin, anhydrojavanicin, bostricoidin, norjavanicin, flaviolin, and 2-hydroxyjuglone) displayed activity against bacteria, yeast, fungi, and plant cells. The mode of action of these molecules was through interference with the host respiration system. By accepting the reducing equivalents from redox enzymes and transferring directly to oxygen, resulting in the respiratory chain being bypassed. In addition, naphthoquinones can act through generation of superoxide radicals and inhibition of glutathione reductases.

## ENZYME AND CLUSTER CROSS-TALK

In addition to the clear interactions between primary and secondary metabolism described above, there are also several examples of cross-talk amongst gene clusters which are responsible for the synthesis of fungal SMs (**Figure 1**). Over-expression of the silent *inp* putative secondary metabolism cross-pathway regulator gene (*scpR*) in *A. nidulans* resulted in the overproduction of both the *inp* gene cluster and also production of the polyketide, asperfuranone. As no link had previously been described between these unrelated metabolites, this work highlighted some additional complexity of regulatory cross-talk in fungal secondary metabolism (Bergmann et al., 2010). Further recent findings are changing previously held views of distinct SM clusters encoding for single class of, or closely related, SMs. A FAD-dependent monooxygenase (FqzB) which is encoded by the fumiquinazoline biosynthetic pathway in *A. fumigatus* was also shown to catalyze spiro-carbon formation in the indole alkaloid spirotryprostatin A via an epoxidation route. The authors speculated that these interactions between unrelated fungal SM-encoding cluster genes may be a strategy of natural product producers to generate structural diversity (Tsunematsu et al., 2013).

### FUMIGACLAVINES AND FUMIQUINAZOLINES

Fumigaclavine C, an ergot alkaloid, was demonstrated to aid in oxidative stress defense in *A. fumigatus* whereby deletion of either of the NRP synthetase genes *pesL* or *pes1* resulted in abrogation of the metabolite (O’Hanlon et al., 2012). Loss of fumigaclavine C in *A. fumigatus* resulted in H_2_O_2_ sensitivity, suggestive of its role in oxidative stress defense. It should be noted that, in contrast to *C. purpurea* (Haarmann et al., 2005), the role of an NRP synthetase in alkaloid synthesis has yet to be confirmed in *A. fumigatus*. Loss of fumigaclavine C was accompanied by elevated levels of another class of SM, the fumitremorgins. The increased production of fumitremorgins such as TR-2, fumitremorgin C, and verruculogen in response to fumigaclavine C diminution is indicative of cross-talk within the secondary metabolome. These authors have speculated that increased fumitremorgin levels are possibly due to increased isoprene availability due to decreased prenylation of fumigaclavine A due to PesL/Pes1 absence. This is supported by the findings of Wiemann et al. (2012), who saw an increase in the isoprene derived molecule gibberellin following loss of either PKS or NRPS activity due to deletion of *ppt1* in *Fusarium fujikuroi.* Interestingly, biosynthesis of fumiquinazolines A through F continues despite the loss of *pesL*, even though this NRP synthetase has been shown to mediate fumiquinazoline biosynthesis *in vitro* (Ames et al., 2010), an observation shared by Lim et al. (2014). This suggests that alternative NRP synthetases can be utilized for fumiquinazoline biosynthesis, which implies cross-talk between SM gene clusters.

### REDOX CONTROL AND GENE CLUSTER MODULATION

O’Keeffe et al. (2014) have surprisingly demonstrated that an intact gliotoxin self-protection mechanism (Scharf et al., 2010; Schrettl et al., 2010), mediated by GliT, is essential to regulate the biosynthesis of apparently unrelated metabolites such as pseurotin A, fumagillin, and fumitremorgins. Specifically, using RNA-seq these authors have demonstrated that gene cluster expression encoding the biosynthesis of helvolic acid, pseurotin A, fumagillin and fumitremorgins is significantly attenuated in *A. fumigatus ΔgliT* upon gliotoxin exposure. Unexpectedly, alterations in the expression of genes involved in siderophore-mediated iron transport and siderophore biosynthesis was also observed, which was suggestive of a deficiency in iron-sensing. Along with alterations in multiple additional cellular systems, these data suggest that GliT-mediated gliotoxin formation is essential to ensure global SM biosynthesis in *A. fumigatus* can occur in a regulated manner, and that cellular redox control, amongst other cellular systems, may play an important role in regulating SM biosynthesis (**Figure 1**).

Further to the observations regarding fumitremorgin and fumigaclavine biosynthesis (O’Hanlon et al., 2012), overexpression of the *A. fumigatus* putative Zn(II)_2_Cys_6_ transcription factor *hasA* [which is part of the hexadehydro-astechrome (HAS) biosynthetic gene cluster] also resulted in the production of large amounts of fumitremorgins compared to the wild-type strain. Deletion of the HAS NRPS in a background overexpressing *hasA* (OE*::hasA*Δ*hasD*) resulted in significantly increased fumitremorgin production. The authors hypothesized that the diversion of tryptophan and prenylation activity from HAS production toward fumitremorgin biosynthesis may occur in OE*::hasA*Δ*hasD* due to the inability of this mutant to utilize the pathway intermediates from HAS production. As fumitremorgins are potent tremorgenic mycotoxins this may account for the increased virulence of the OE*::hasA*Δ*hasD* strain compared to wild-type (Yin et al., 2013). This degree of metabolite cross-talk has one limiting affect, because it infers that caution must be exercised in interpreting the impact of metabolite-altering gene deletion experiments. In other words, if metabolite one disappears, and the level of an apparently unrelated metabolite two elevates – which change is altering phenotype?

Although HAS does not contribute to the uptake of extracellular siderophores or iron detoxification in *A. fumigatus*, an iron starvation phenotype and concomitant siderophore biosynthesis is induced by HAS or astechrome (Wiemann et al., 2014). It has now been demonstrated that iron levels regulate the biosynthesis of HAS, and other SM, which is dependent upon the bZIP protein, HapX and the GATA-type transcription factor, SreA. Wiemann et al. (2014) have also demonstrated that HAS not only influences iron acquisition but also the expression of multiple gene clusters involved in SM biosynthesis. They further speculate that intracellular levels of iron (and HAS) may trigger, at a systems level, primary metabolite availability to effect SM biosynthesis, in a redox-dependent manner. These contemporaneous observations are complementary to those of O’Keeffe et al. (2014) and suggest that we are beginning to piece together the systems interactions, via SM cross-talk, necessary to facilitate SM biosynthesis in this human opportunistic pathogenic fungus (**Figure 1**).

### EFFLUX

The eﬄux of SMs is another aspect of secondary metabolism where evidence exists of the occurrence of cross-metabolite interactions. The rice pathogen *Magnaporthe grisea* produces the PKS–NRPS fusion natural product ACE1 from a 15-gene cluster. Although this cluster encodes a transporter for the MFS superfamily (MFS1), it has been shown that *MFS1* is not involved in the eﬄux of ACE1 as this gene has a deletion of a single base pair which results in an early stop codon. Therefore, it has been suggested that ACE1 must rely on another transporter which is encoded outside of the ACE1 gene cluster (Coleman and Mylonakis, 2009). Moreover, deletion of the sirodesmin ABC transporter gene *sirA* from *Leptosphaeria maculans* actually resulted in an increase (39%) of sirodesmin production and secretion compared to the wild-type strain. The production of deacetyl sirodesmin in the Δ*sirA* mutant also increased 27% compared to the wild-type. This seemingly contradictory result may be explained by the presence of alternate eﬄux mechanisms for these metabolites or a degree of redundancy across SM transporters. These alternate eﬄux mechanisms may be more effective than SirA, resulting in differential sirodesmin cluster feedback regulation which leads to the overexpression of this NRPS cluster (Gardiner et al., 2005b). In contrast, deletion of *gliA* from the gliotoxin gene cluster significantly decreased gliotoxin eﬄux, which indicates that some ETP-producing fungi may not have compensatory mechanisms to mediate natural product eﬄux (Wang et al., 2014). Evidence also exists to suggest that the MFS transporter DotC encoded within the dothistromin gene cluster of *Dothistroma septosporum* is not the only mechanism of toxin eﬄux in this organism (Bradshaw et al., 2009).

### INTERTWINED GENE CLUSTERS

The *A. fumigatus* genes *psoF* (putative dual function methyltransferase and monooxygenase) or *psoG* (hypothetical protein) were predicted to be required for fumagillin biosynthesis due to their proximity to the characterized fumagillin encoding genes *fmaA* and *fmaB*. Surprisingly, deletion of *psoF* and *psoG* resulted in the abolition of pseurotin A biosynthesis. The Δ*psoF* strain accumulated a demethyl-deepoxy-synerazol (*m/z* 384.1447) compound instead of pseurotin A which is in agreement with the putative role of this enzyme (Wiemann et al., 2013). PsoF was recently characterized by Tsunematsu et al. (2014). This work highlighted an additional layer of complexity regarding the interactions between SMs and is undoubtedly one of the most dramatic examples of cross-talk between fungal metabolites, at the genetic level, which has been elucidated by Wiemann et al. (2013) in *A. fumigatus*. Here, the gene clusters encoding fumagillin and the NRP/polyketide hybrid, pseurotin A, are physically intertwined and co-regulated by LaeA via the Zn(II)_2_Cys_6_ Transcription Factor, FapR. In addition, fumitremorgin is also encoded by this supercluster; however, the genes encoding the biosynthesis of this metabolite are distinct from the intertwined region. Interestingly, although this supercluster is not present in completely intact form in related fungal species, there is sufficient co-localization of orthologs to allow Wiemann et al. (2013) to speculate that co-production of the aforementioned metabolites confers survival advantages on producing species. It is tempting to speculate that the products of this supercluster act synergistically or in a complementary manner, almost like subunits of a heteromeric enzyme, to effect survival of *A. fumigatus*, and related species in defined ecological niches.

It is also true to say that hybrid PKS-NRPS megasynthetases represent a unique concept in metabolite cross-talk, whereby PK and NRPs are reconstituted into unique molecular entities and this topic has been extensively reviewed elsewhere (Boettger and Hertweck, 2013).

### BEYOND ENDOGENOUS: INTER-KINGDOM CROSS-TALK

Recent data have also described what may be cross-talk of natural products from different organisms. Culturing *A. fumigatus* MBC-F1-10 in the presence of *Streptomyces bullii* leads to the production of a diversity of *A. fumigatus* metabolites including ergosterol and seven diketopiperazine (DKP) class of alkaloids. Production of the antibiotic–antitumor metabolite glionitrin was induced in *A. fumigatus* following co-culture with a *Sphingomonas* isolate KMK-001 derived from mine-drainage system (Rateb et al., 2013). Moreover, physical interaction between the *S. rapamycinicus* and *A. fumigatus* resulting in the activation of a silent PKS gene cluster encoding fumicycline A (König et al., 2013). Bacterial metabolites have also been demonstrated to act as precursors of fungal metabolites, whereby phenazine metabolites from *Pseudomonas aeruginosa* were converted by *A. fumigatus* into new molecular species with enhanced toxicity, as well as additional, properties (Moree et al., 2012). It is likely that further examples of inter-kingdom interactions, which describe cross-talk leading to metabolite production, will be described as increasing attention is being focused on the impacts of co-culturing of microorganisms.

## CONCLUDING REMARKS

Upon writing this manuscript, the authors could not help but be struck once again by two terms which continuously pervade work on fungal natural products – these are NRPS and *secondary* metabolism. In other words, we ask the question if research in and funding for, this exciting area of chemical biology is hindered by the use of the negative terminology of *non* and *secondary*? Perhaps it is time to describe the biosynthesis of these peptidyl entities in more positive ways, and not to refer to the processes of bioactive metabolite formation as a *secondary*, or somehow less important or optional, system. It is not – evolution generally sees to that! Perhaps CMPS (Cluster Mediated Peptide Synthesis) could replace NRPS, as it simultaneously (i) removes the negative terminology of *non*, (ii) increases the descriptive value of the acronym to suggest *gene cluster* involvement in the process, and (iii) offers the possibility of referring to NRPs as or CDPs (Cluster Peptides or Cluster Derived Peptides).

So, it is undoubtedly clear that significant cross-talk exists between (i) primary and secondary metabolism, (ii) different fungal metabolites, (iii) the enzymes involved in the synthesizing different metabolites, and (iv) various metabolite-encoding gene clusters (**Figure 1**). Indeed cross-talk even exists between kingdoms. As described herein, and elsewhere (Lim and Keller, 2014), the details, significance and potential of this exquisite orchestration of unforeseen molecular events are emerging at an ever-increasing rate. For the future, high-throughput analytical approaches, combined with new insights into, and revised conceptions of, fungal genetics and biochemistry, will yield further surprises which will continue to enthuse us to re-imagine our current perspective of distinct orders of cellular metabolic processes in these truly unique organisms.

## Conflict of Interest Statement

The authors declare that the research was conducted in the absence of any commercial or financial relationships that could be construed as a potential conflict of interest.
